# Supplementary material for: The unique allosteric property of crocodilian haemoglobin elucidated by cryo-EM
Source: Nat Commun. 2024 Aug 2;15:6505. doi: 10.1038/s41467-024-49947-x (PMC11294572; doi:10.1038/s41467-024-49947-x)
Supplement: Supplementary file 1 — Supplementary Information [file 41467_2024_49947_MOESM1_ESM.pdf]

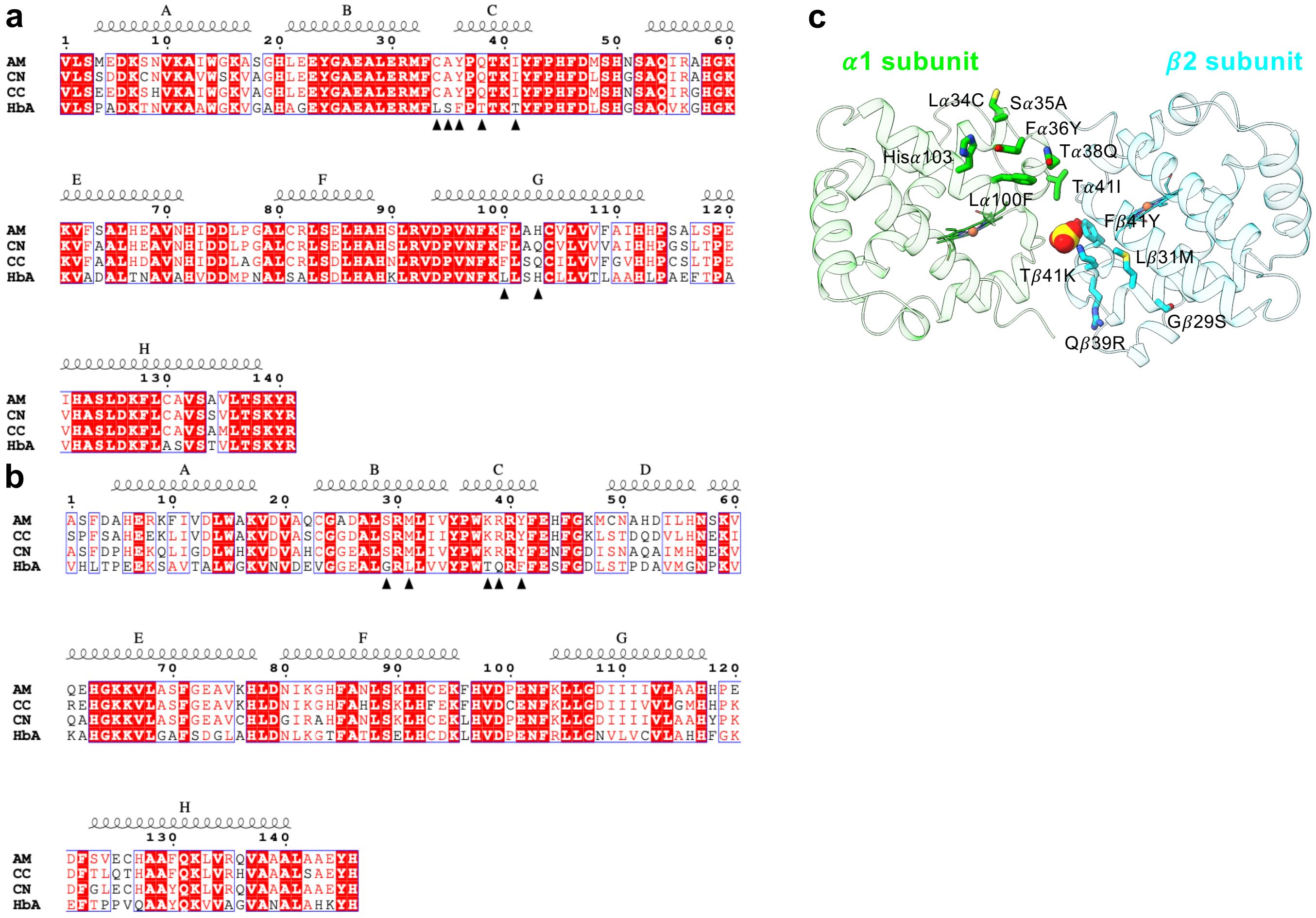

Figure S1. Sequence comparison of human and crocodilian Hb. (**a**) Alignment of human α globin with the homologous protein from three crocodilian species: AM (*Alligator mississipiensis*), CN (*Crocodylus niloticus*), and CC (*Caiman crocodilus*). Amino acid residues in common are shown in white on red; conservative mutations are shown in red. The traditional naming scheme of helical regions is shown above the sequences. Black triangles indicate residues mutated to create Hb Scuba. (**b**) Alignment of β globin sequences. These figures were made with ESPRIPT[1]. (**c**) Residues mutated to create Hb Scuba are shown as sticks in a ribbon model of deoxy HbAM, showing the $\alpha_1\beta_2$ subunits. His α103 is the only residue common to human HbA and HbAM at these 12 positions. The α_1 subunit is shown in lime and the β_2 subunit in cyan. The bicarbonate ion is shown as a CPK model, with carbon yellow and oxygen red.

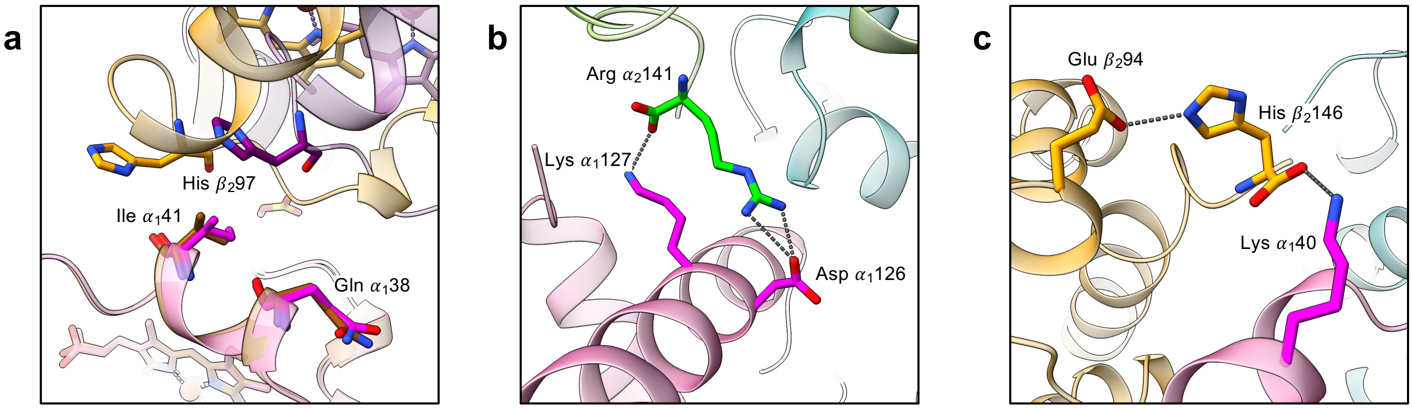

Figure S2. Conserved features of HbAM. (**a**) The movement of His $\beta_2$97 relative to the α_1 C helix. The carbon atoms of the α_1 subunit are shown in magenta, of the α_2 subunit in cyan, of the β_1 subunit in lime, and of the β_2 subunit in orange. (**b**) Asp $\alpha_1$126 and Arg $\alpha_2$141. (**c**) Lys $\alpha_1$40, His $\beta_2$146 and Glu $\beta_2$94.

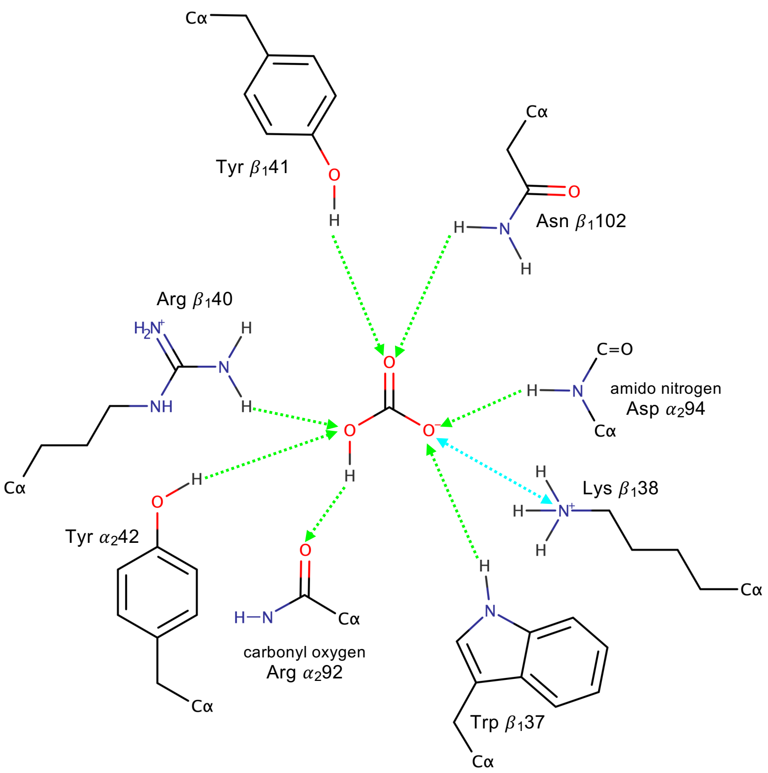

Figure S3. Schematic diagram of the bicarbonate binding site. Hydrogen bonds are shown as lime colored arrows, and a salt-bridge is shown as a two-headed arrow colored cyan. The carbonyl oxygen of Arg α92 accepts a hydrogen bond from the bicarbonate ion.

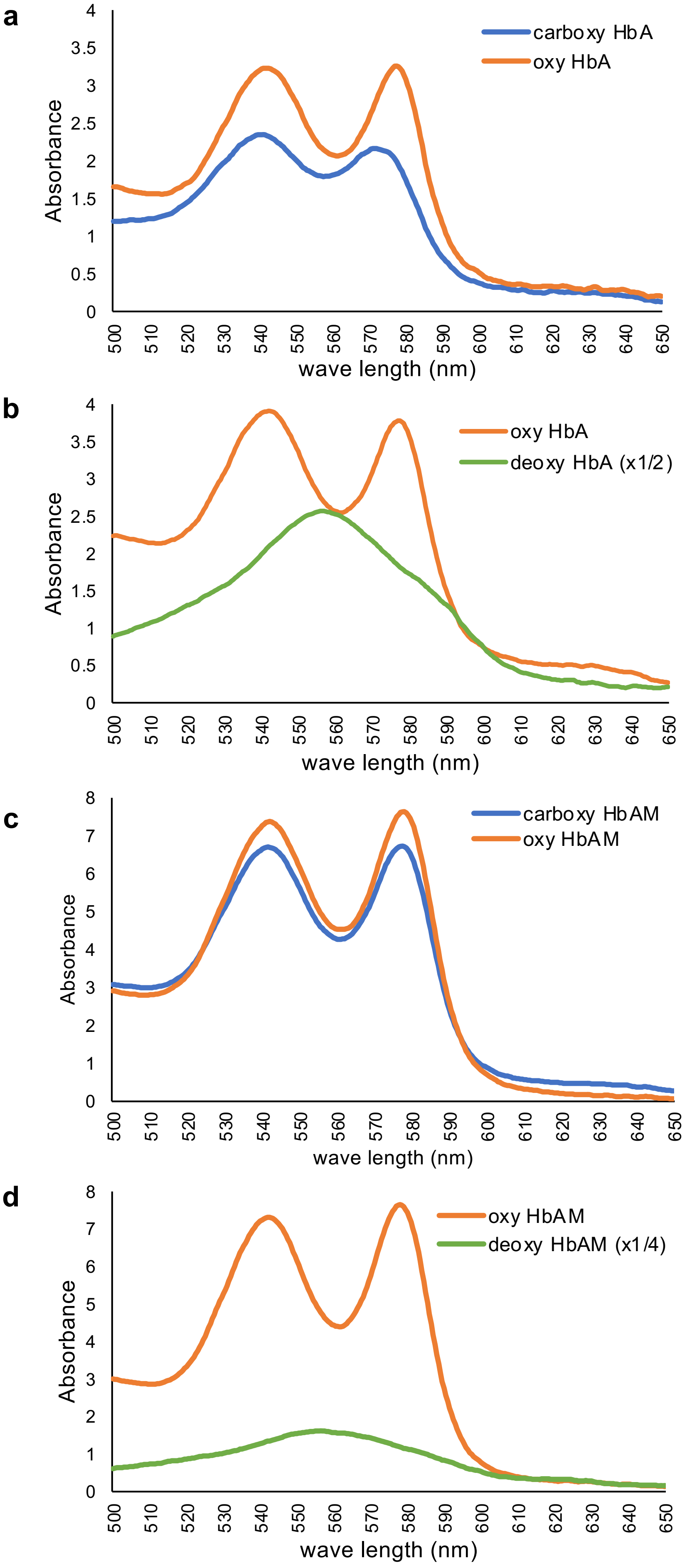

Figure S4. Visible spectra of the proteins. (**a**) Carbonmonoxy (blue) and oxy (orange) human HbA, measured under air before vitrification. (**b**) Oxy human HbA measured under oxygen-free nitrogen in the anaerobic chamber, before (orange) and after (green) addition of dithionite. (**c**) carbonmonoxy and oxy HbAM. (**d**) Oxy HbAM, measured in the anaerobic chamber before (orange) and after (green) addition of dithionite.

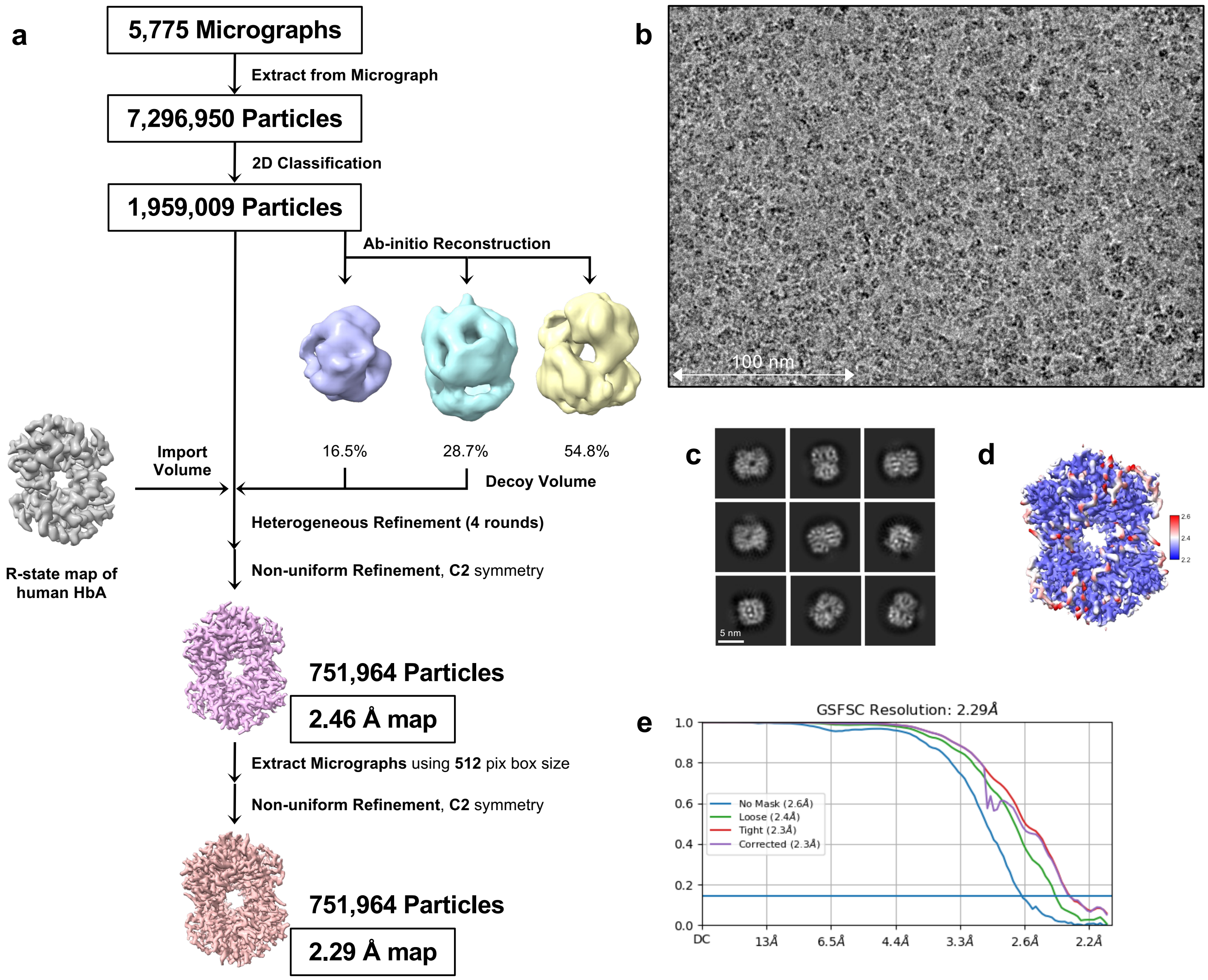

Figure S5. Structural analysis of carbonmonoxy HbAM. (**a**) Data processing workflow. (**b**) Representative cryo-EM micrograph. (**c**) Representative 2D class averages. (**d**) Local resolution estimation of the final map. (**e**) Gold-standard Fourier shell correlation plot.

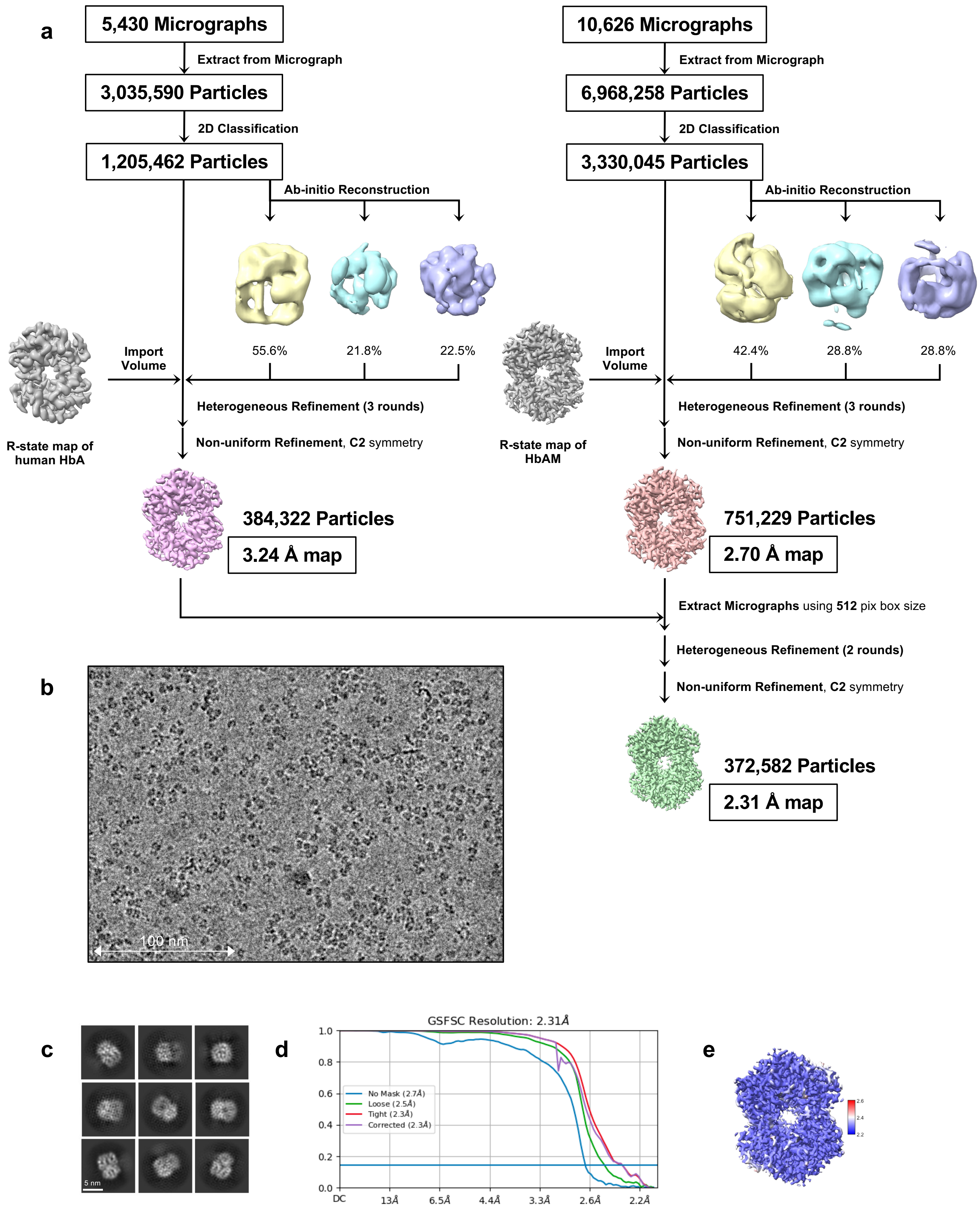

Figure S6. Structural analysis of oxy HbAM. (**a**) Data processing workflow. (**b**) Representative cryo-EM micrograph. (**c**) Representative 2D class averages. (**d**) Gold-standard Fourier shell correlation plot. (**e**) Local resolution estimation of the final map.

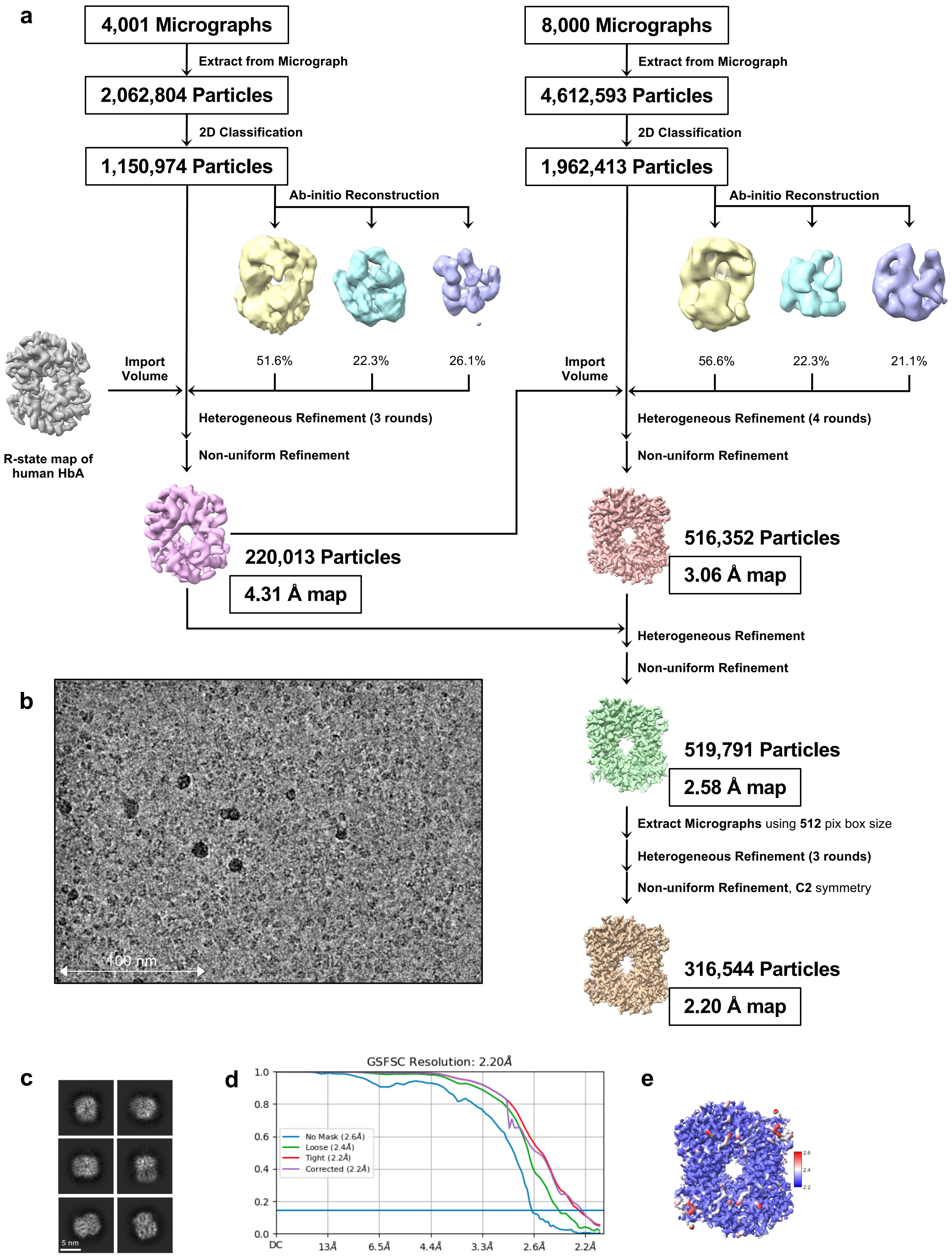

Figure S7: Structural analysis of deoxy HbAM. (**a**) Data processing workflow. (**b**) Representative cryo-EM micrograph. (**c**) Representative 2D class averages. (**d**) Gold-standard Fourier shell correlation plot. (**e**) Local resolution estimation of the final map.

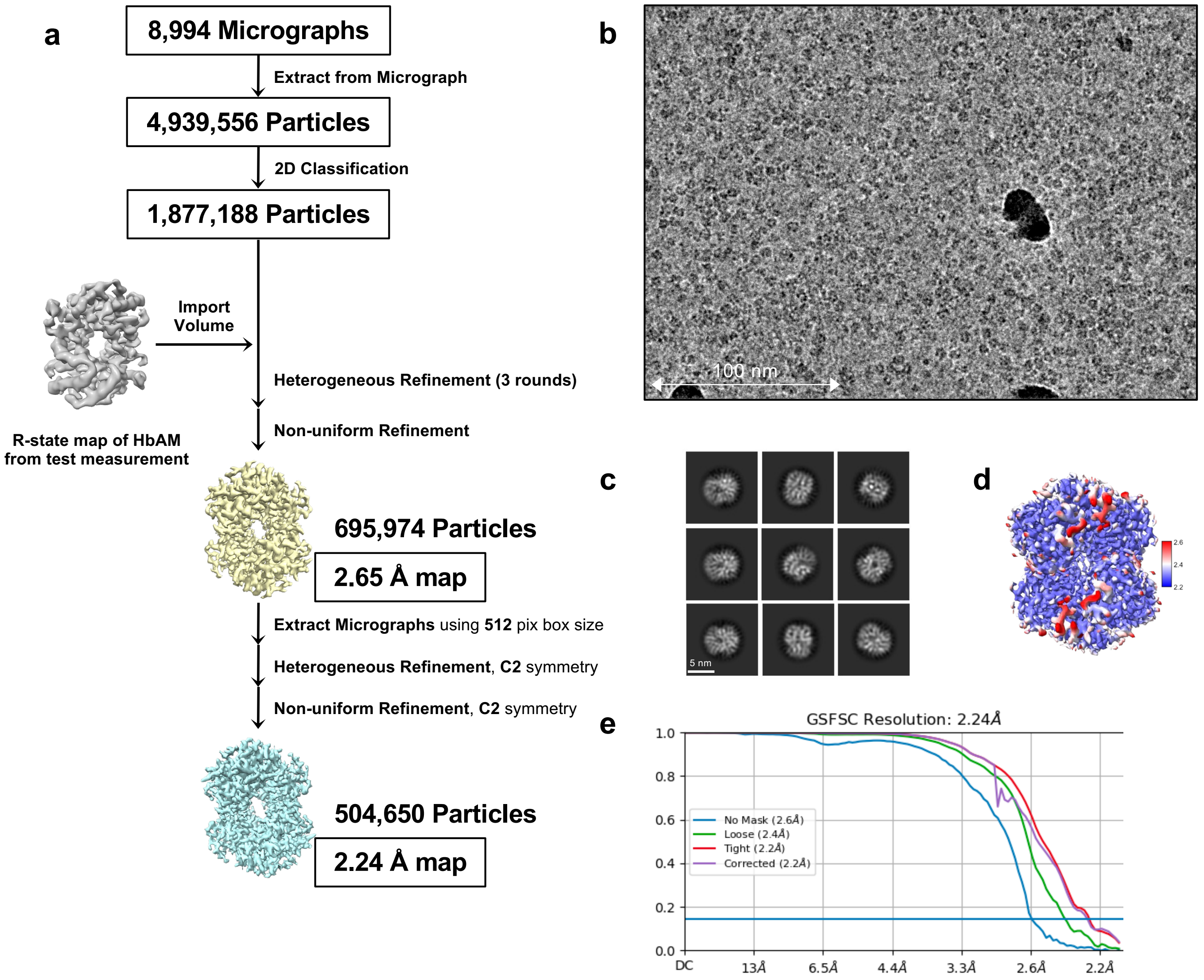

Figure S8. Structural analysis of carbonmonoxy human HbA. (**a**) Data processing workflow. (**b**) Representative cryo-EM micrograph. (**c**) Representative 2D class averages. (**d**) Local resolution estimation of the final map. (**e**) Gold-standard Fourier shell correlation plot.

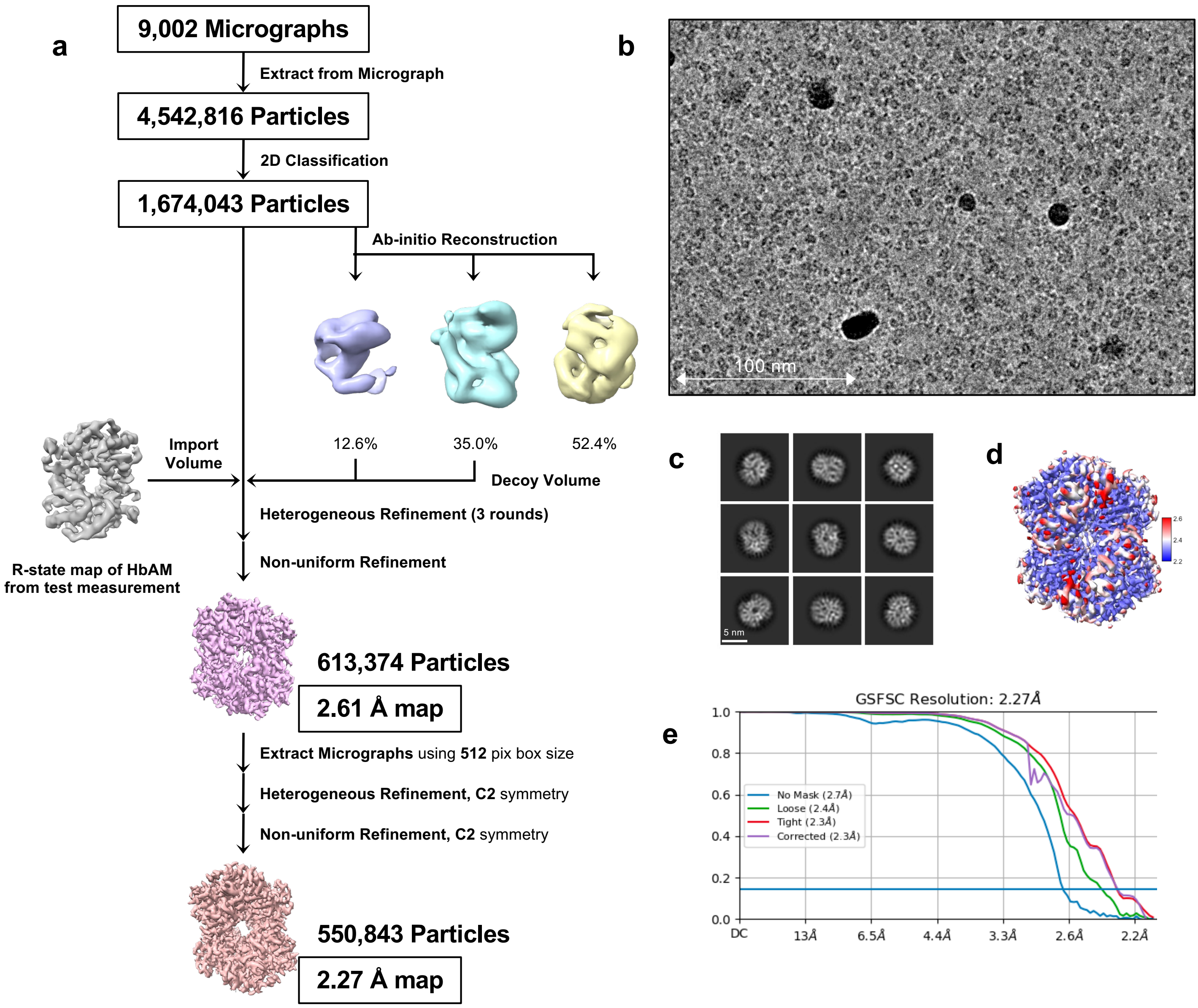

Figure S9. Structural analysis of oxy human HbA. (**a**) Data processing workflow. (**b**) Representative cryo-EM micrograph. (**c**) Representative 2D class averages. (**d**) Local resolution estimation of the final map. (**e**) Gold-standard Fourier shell correlation plot.

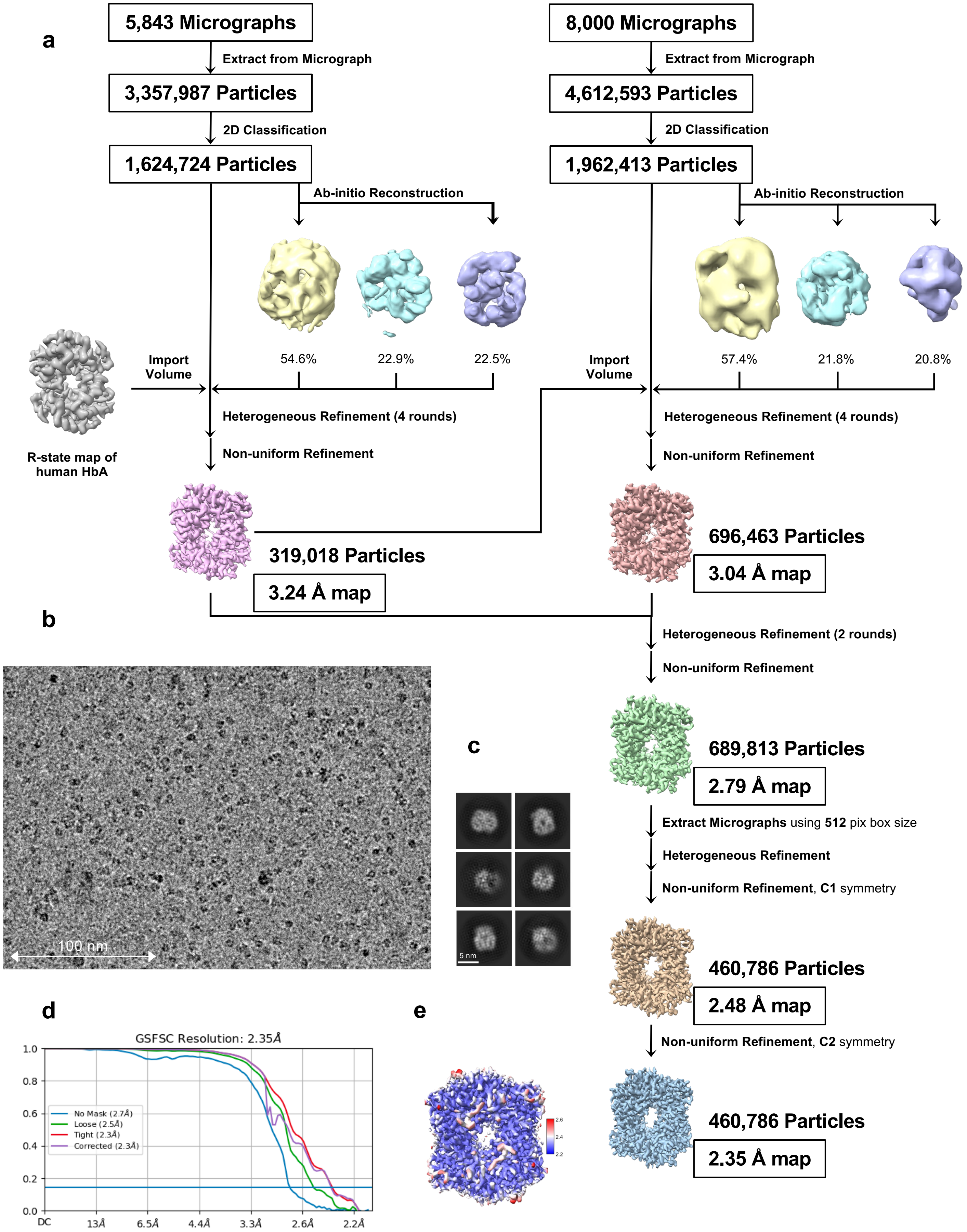

Figure S10. Structural analysis of deoxy human HbA. (**a**) Data processing workflow. (**b**) Representative cryo-EM micrograph. (**c**) Representative 2D class averages. (**d**) Gold-standard Fourier shell correlation plot. (**e**) Local resolution estimation of the final map.

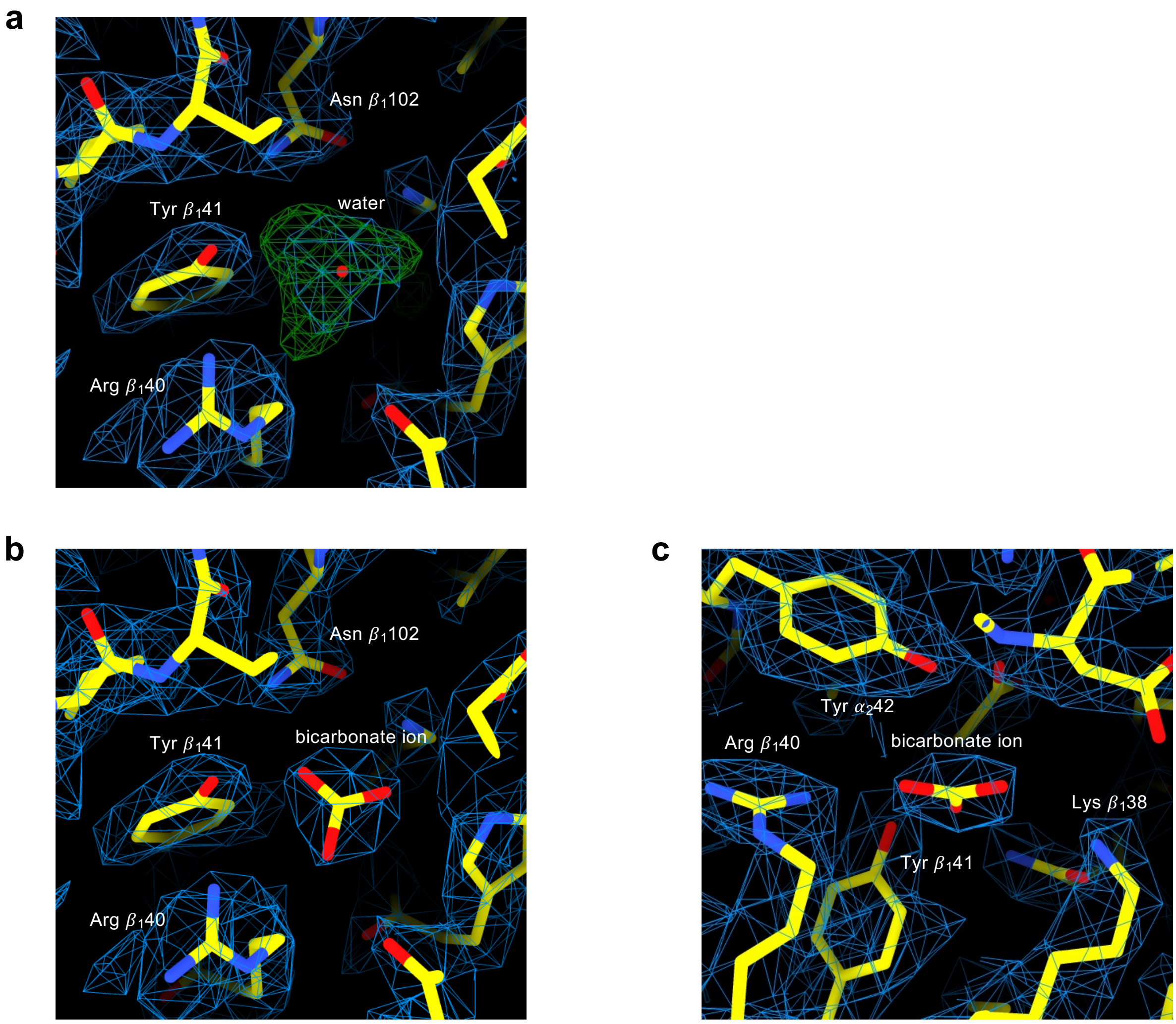

Figure S11. Density maps at the bicarbonate binding site. (**a**) The water molecule initially placed at this position (red sphere) is shown in the cryo-EM density map (blue) and the F_O-F_C positive map (green), which indicates a larger, non-spherical ligand. (**b, c)** The bicarbonate ion in the final model and the cryo-EM density map (blue) from two different angle.

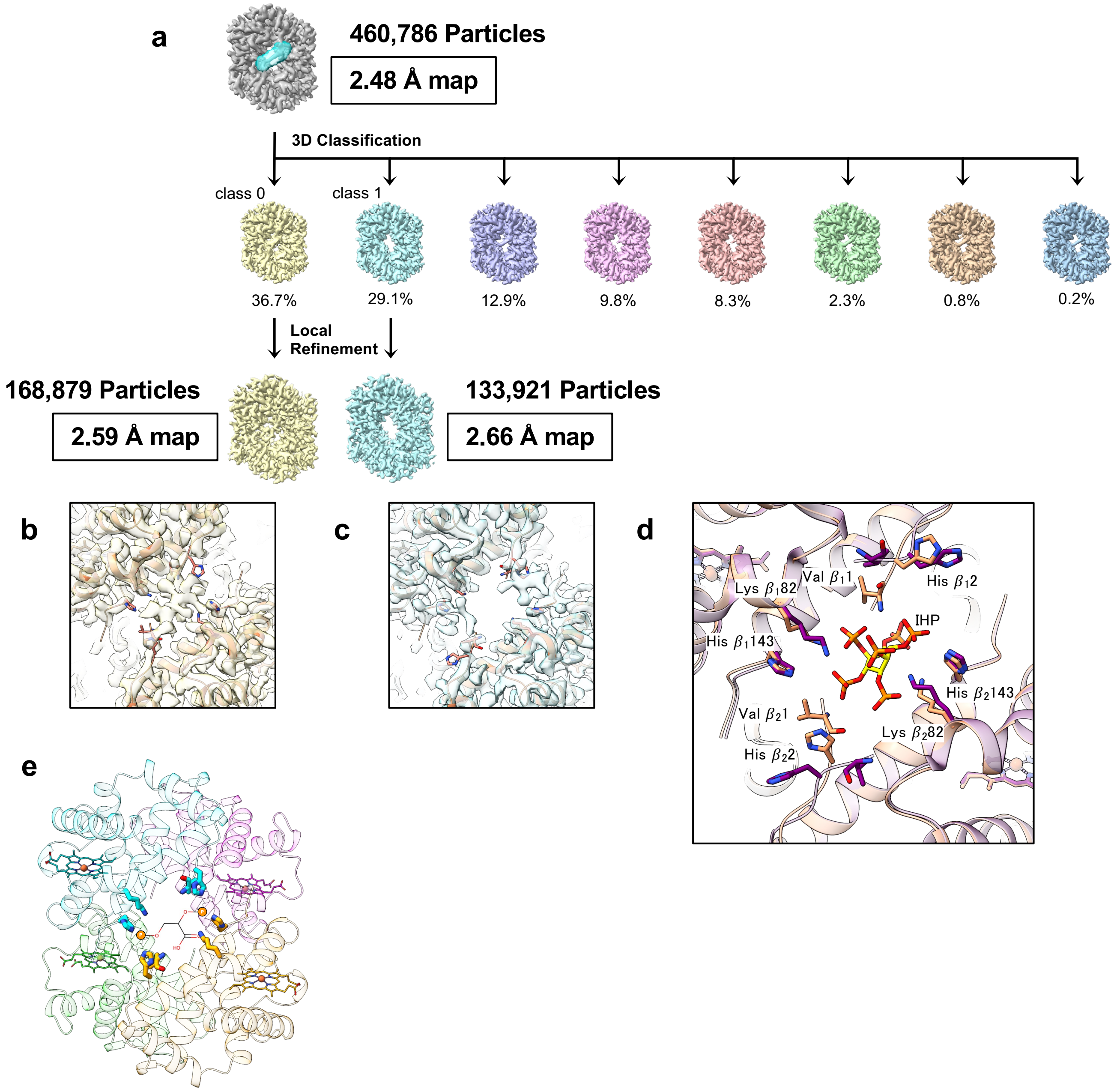

Figure S12. 3D classification of deoxy human HbA. (**a**) Workflow of 3D classification and local refinements. A mask around the IHP-like blob is shown in cyan. The particles were classified into eight classes (class 0-7, from left to right), and the maps of class 0 (yellow) and class 1 (light blue) were refined respectively. Close-up of the phosphate interacting site between two β subunits in the cryo-EM map of IHP-bound deoxy human HbA (**b**) and unliganded deoxy human HbA (**c**) generated from local refinement. (**d**) A possible conformation of IHP between the two β subunits of deoxy human HbA, superposed with carbonmonoxy human HbA. The carbon atoms of deoxy human HbA are colored salmon, those of carbonmonoxy human HbA are shown in purple, and those of IHP are shown in yellow. (**e**) A hypothetical model of the 2,3-DPG (diphosphoglycerate)-bound deoxy human HbA[8,9], with interacting side-chains shown as the sticks model. The carbon atoms of the α_1 subunit are shown in magenta, those of the β_1 subunit are shown in cyan, those of the α_2 subunit are shown in lime, and those of the β_2 subunit are shown in orange. 2,3-DPG is shown as the structural formula, and phosphate groups are shown as orange circles.

Table S1. Distances between atoms of ligands, heme, and histidines of the α subunit of carbonmonoxy human HbA and HbAM (**a**), and oxy human HbA and HbAM (**b**).

a

**distance (Å)**		**2dn3**	**1bbb**	**CO-HbA**	**CO-HbAM**	**ideal**
CO - C	HEM - Fe	1.74	1.77	1.88	1.98	1.75
CO - C	HEM - Nα	2.72	2.83	2.69	2.78	
CO - O	His α58 - Nε	3.30	3.23	3.02	2.57	
CO - C	His α58 - Nε	3.44	3.47	2.91	2.72	
HEM - Fe	His α87 - Nε	2.11	2.08	2.19	2.19	

b

**distance (Å)**		**2dn1**	**O_2-HbA**	**O_2-HbAM**	**ideal**
O_2 - O_{near}	HEM - Fe	1.82	2.07	2.01	1.75
O_2 - O_{near}	HEM - Nα	2.71	3.03	2.97	
O_2 - O_{near}	His α58 - Nε	2.82	2.57	2.69	
O_2 - O_{far}	His α58 - Nε	2.70	2.95	2.85	
HEM - Fe	His α87 - Nε	2.07	2.15	2.19	

Table S2. Interaction distances of the principal allosteric contacts of carbonmonoxy human HbA and HbAM (**a**), and deoxy human HbA and HbAM (**b**).

a

distance (Å)		2dn3	1bbb	CO-HbA	CO-HbAM
Asp $\alpha_1$94 - Oδ	Trp $\beta_2$37 - Nε	3.67	3.77	3.64	3.38
Asp $\alpha_1$94 - Oδ	Asn $\beta_2$102 - Nδ	2.74	2.75	2.97	2.92
Trp $\beta_1$37 - Nε	Asn $\beta_1$102 - Nδ	2.95	3.03	2.99	3.02

b

distance (Å)		2dn2	Deoxy HbA	Deoxy HbAM
Lys $\alpha_1$40 - Nζ	His $\beta_2$146 - O	2.83	3.00	2.72
Tyr $\alpha_1$42 - Oη	Asp $\beta_2$99 - Oδ	2.51	3.02	2.52
Asp $\alpha_1$94 - Oδ	Trp $\beta_2$37 - Nε	2.85	2.98	3.42
Asp $\alpha_1$126 - Oδ	Arg $\alpha_2$141 - Nη_1	2.78	2.78	2.66
	Arg $\alpha_2$141 - Nη_2	2.95	2.79	3.42
Lys $\alpha_1$127 - Nζ	Arg $\alpha_2$141 - O	2.77	3.68	2.66
Asp $\beta_1$94 - Oδ	His $\beta_1$146 - Nε	2.60	3.09	2.79

Table S3 RMSD comparisons between X-ray and cryo-EM models of all states of human HbA and HbAM (**a, b**).

a

RMSD (Å)	2dn1	2dn3	1bbb	Oxy HbA	CO-HbA	Oxy HbAM
**Oxy HbA**	2.14	1.44	0.61			
**Carbonmonoxy HbA**	2.16	1.46	0.58	0.21		
**Oxy HbAM**	1.26	1.18	1.74	1.47	1.47	
**Carbonmonoxy HbAM**	1.23	1.15	1.75	1.46	1.47	0.15

b

RMSD (Å)	2dn2	Deoxy HbA
**Deoxy HbA**	0.44	
**Deoxy HbAM**	0.99	0.84

	angle (°)
2dn2	0
Deoxy human HbA	1.3
Deoxy HbAM	3.1
2dn1 (Oxy)	14.7
Oxy HbAM	15.0
2dn3 (CO)	15.0
Carbonmonoxy HbAM	16.4
Oxy human HbA	19.9
Carbonmonoxy human HbA	22.1
1bbb	23.8

Table S4. Concerted rotation angles with respect to 2dn2 (X-ray model of the T-state deoxy human HbA) of all state structures of human HbA and HbAM. The colours of the underlines on the left column match the colours of the aligned models in Figure 2.

Residue	Distance (Å)
Trp β37 - Nε	3.32
Lys β38* - Nζ	2.77
Arg β40 - Nη	2.94
Tyr β41* - Oη	2.36
Asn β102 - Nδ	2.98
Tyr α42 - Oη	3.34
Arg α92 - $O_{carbonyl}$	2.68
Asp α94 - N_{amido}	3.44

Table S5. Interaction distances between bicarbonate ions and deoxy HbAM. The asterisks show the unique crocodilian residues directly binding bicarbonate.
